# Vitamin D Supplementation and Impact on Skeletal Muscle Function in Cell and Animal Models and an Aging Population: What Do We Know So Far?

**DOI:** 10.3390/nu13041110

**Published:** 2021-03-28

**Authors:** Karina Romeu Montenegro, Milene Amarante Pufal, Philip Newsholme

**Affiliations:** 1 Curtin Medical School, Curtin Health Innovation Research Institute, Curtin University, Perth, WA 6102, Australia; philip.newsholme@curtin.edu.au; 2 Department of Nutrition, MD Dermatology, Porto Alegre, Rio Grande do Sul 90880-481, Brazil; milene.pufal@gmail.com

**Keywords:** skeletal muscle function, vitamin D, aging, muscle cells, calcitriol

## Abstract

Aging is associated with impairment in skeletal muscle mass and contractile function, predisposing to fat mass gain, insulin resistance and diabetes. The impact of Vitamin D (VitD) supplementation on skeletal muscle mass and function in older adults is still controversial. The aim of this review was to summarize data from randomized clinical trials, animal dietary intervention and cell studies in order to clarify current knowledge on the effects of VitD on skeletal muscle as reported for these three types of experiments. A structured research of the literature in Medline via PubMed was conducted and a total of 43 articles were analysed (cells *n* = 18, animals *n* = 13 and humans *n* = 13). The results as described by these key studies demonstrate, overall, at cell and animal levels, that VitD treatments had positive effects on the development of muscle fibres in cells in culture, skeletal muscle force and hypertrophy. Vitamin D supplementation appears to regulate not only lipid and mitochondrial muscle metabolism but also to have a direct effect on glucose metabolism and insulin driven signalling. However, considering the human perspective, results revealed a predominance of null effects of the vitamin on muscle in the ageing population, but experimental design may have influenced the study outcome in humans. Well-designed long duration double-blinded trials, standardised VitD dosing regimen, larger sample sized studies and standardised measurements may be helpful tools to accurately determine results and compare to those observed in cells and animal dietary intervention models.

## 1. Introduction

The evidence for prolonged aging in the human population is becoming clear. It has been predicted that within the next 3 decades, more than 20% of the US population will be 65 years old or older [1]. In addition, this older population is at increased risk of developing vitamin D (VitD) insufficiency {levels of serum 25-hydroxyvitamin D [25(OH)D] below 50 nmol/L (20 ng/mL) due to the following main factors: declination of skin’s ability to synthesize VitD, reduced sun exposure as they tend to spend more time indoors [2] coupled with insufficient intake of the vitamin from food [3].

The process of aging is normally associated with a reduction in muscle mass, function and also with the development of frailty, which significantly reduces life expectancy [4,5,6]. The subsequent loss of skeletal muscle mass and strength is known as sarcopenia [5]. Metabolic diseases are generally part of this scenario, negatively impacting on skeletal muscle tissue [7]. However, studies indicate that there may be a variation across the population in relation to the rates of losing muscle mass over the years suggesting that diet and lifestyle may play a powerful influence on this process [8]. Different hormones and nutrients have been reported to influence skeletal muscle mass and VitD has been suggested to be one of them. So, it seems that the role of VitD lays beyond the bone and mineral metabolism as studies have been suggesting that supplementation with VitD has a potential positive effect on skeletal muscle function [9,10]. 

In cellular and animal models, a wide range of mechanisms by which VitD may impact skeletal muscle function has been suggested. The majority of studies in this area have been investigating the effects of VitD3 in aging process, mainly due to the reasons cited above. They include measurements of skeletal muscle mass, strength and function, oxidative stress, fat metabolism, mitochondrial function, insulin sensitivity, GLUT 4 regulation, protein synthesis, myotube formation/fibres and advanced glycation end-products (AGES) [11,12,13,14,15,16,17]. As there is a vast range of pathways and metabolism influenced by Vitamin D, here we will focus and elucidate the possible effects of VitD3 supplementation on skeletal muscle function limited to the aging process.

To date, the literature reports an association of some health conditions such as obesity and type 2 diabetes (T2DM) and indirectly glucotoxicity and lipotoxicity [11] with VitD deficiency. Skeletal muscle is the predominant insulin target tissue and plays a key role in regulating glucose uptake, metabolism and storage [7,18] which optimal function is vital for exercise, glucose and amino acid metabolism. In addition, many studies have confirmed that skeletal muscle cells express the VitD receptor (VDR)-which normally decreases with age and may increase after VitD supplementation [1]. Briefly, 1,25 dihydroxyvitamin D [1,25(OH)2D] binds to the nuclear VDR and forms a complex with the retinoid receptor (RXR).1,25(OH)2D/VDR/RXR complex then modulates the transcription of various genes, but also participates on pathways leading to non-genomic effects [19]. In this sense, VDR activation would promote muscle protein synthesis [20] by enhancing the stimulus effect of leucine and insulin on protein synthesis in murine C2C12 myotubes in a dose-dependent manner. Furthermore, it is possible that VitD3 supplementation would also enhance mitochondrial function and would consequently optimize contractility through regulation of energy production and calcium and phosphate levels [21]. It has been reported that VitD3 impacts in committed myoblasts depending on the cell model, maturation, treatment dose, duration, cell origin and species [22]. 

Translating what we have previously described above to a human level, there is a growing support for the potential benefits of VitD3 supplementation regarding increase in muscle mass or strength and function in elderly people [23]. Studies suggest a possible favourable role of VitD supplementation in people with deficiency of total serum [25(OH)D] levels on muscle strength [24]. However, there is no consensus on this possibility mainly due to contradictory outcomes [25]. If reported to be effective, this treatment could be carried out rapidly and it could be inexpensively incorporated into healthcare services not only to bring total serum [25(OH)D] levels into normality but also to improve bone and muscle health in this specific population. 

As observation studies cannot prove causality, we aimed to review data from randomized clinical trials, animals and cell studies in order to clarify what we know so far about the effects of VitD on skeletal muscle at these three levels of experiments.

## 2. Methods

### 2.1. Search Strategy

We conducted a structured research of the literature on the 26 December 2020 and reviewed on the 10 February 2021 in Medline via PubMed using the following keywords (and each one’s MeSh terms): “Vitamin D”; “Skeletal muscle”; “Children”; “Pregnancy”; “Breastfeeding”; “Adolescent”. Boolean operators (AND, OR, NOT) were used in order to create a focused search strategy.

PRISMA search method: ((“Vitamin D” OR “cholecalciferol” OR “ergocalciferol” OR “1,25-dihydroxyvitamin” OR “25-hydroxy-Vitamin D” OR “calcitriol”) AND (“Skeletal muscle” OR “Muscle Cells” OR “Fiber, Skeletal Muscle” OR “Skeletal Muscle Fiber” OR “Skeletal Myocytes” OR “Skeletal Muscle Fibers” OR “Myocytes, Skeletal” OR “Skeletal Myocyte” OR “Myotubes” OR “Myotube” OR “Muscle Fibers, Fast-Twitch” OR “Fast-Twitch Muscle Fibers” OR “Myoblasts” OR “Myoblast” OR “Skeletal Myoblast” OR “Skeletal Myoblasts”)) NOT (“children” OR “child” OR “preschool child” OR “preschool children” OR “infant” OR “pregnancy” OR “gestation” OR “pregnant woman” OR “pregnant women” OR “breastfeeding” OR “adolescent” OR “teen” OR “teenager” OR “youth” OR “minor”). 

### 2.2. Inclusion Criteria

We included only intervention studies that evaluated the effects of VitD supplementation alone on skeletal muscle parameters. Participants were considered in an aging stage of life by the authors, who decided to include participants with 50 years or more of age. The supplementation could have been with any source of VitD and the control group had to be supplemented with placebo in the various studies. Humans, animals and cells studies were eligible if they were published from 2000 to November 2020.

### 2.3. Exclusion Criteria 

**Figure 1:**
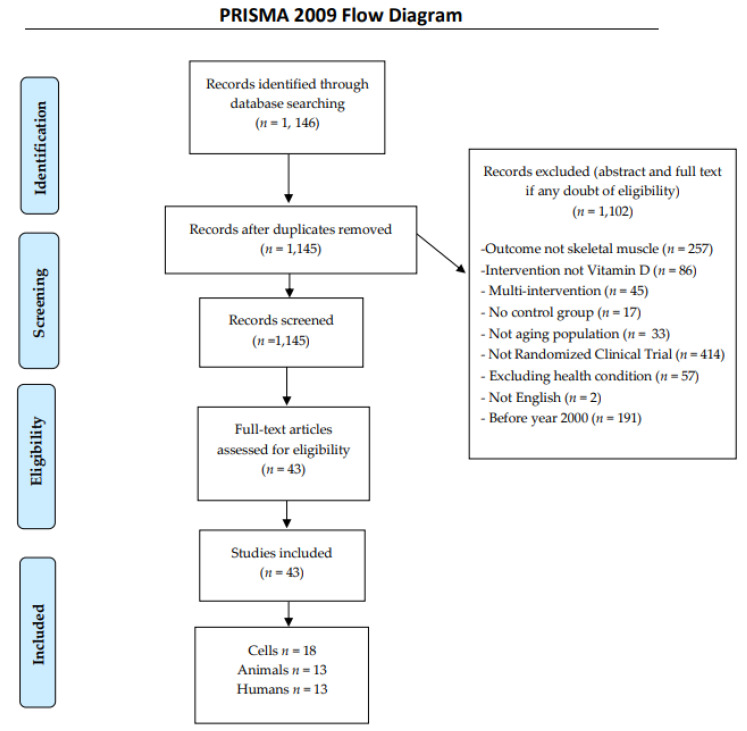
PRISMA flow diagram of research stages.

Intervention with multiple components were not included (for instance: VitD supplementation associated with protein shake or any other macro or micronutrient or medicine prescription). In addition, participants should not have any conditions that could affect food intake or absorption [digestive disorder (e.g., irritable bowel syndrome, inflammatory bowel disease)] or any condition that prevented people from chewing or swallowing (e.g., edentulous) or any gastrointestinal tract procedure that permanently affected absorption (e.g., bariatric surgery), eating disorder (e.g., anorexia, bulimia), significant/chronic disease (e.g., HIV, cancer–cardiovascular conditions; active, chronic respiratory failure, Alzheimer disease, Parkinson disease, drug/alcohol dependence) or early post-surgery (Figure 1).

### 2.4. Data Extraction

Figure 1 describes the flowchart of the research stages. The initial research was made independently by the two first authors. (KRM and MAP). After excluding papers according to the criteria, a total of 43 studies were then analysed. Two reviewers worked independently (KRM and MAP). One author (KRM) extracted data from animal and cell studies whereas the other (MAP) collected data from human trials. Revision of the manuscript draft was made by a third author (PN). Any questions about any data were discussed between authors. Table 1, Table 2 and Table 3 report the extracted data from each included study in cells, animals and humans, respectively.

## 3. Results and Discussion

### 3.1. Cell Lines 

Seven cell lines studies (Table 1) were identified in this review. Eleven out of the 18 studies used C2C12 murine cells, while three studies have used human muscle cells and only one study have used each of the following cell lines: L6, mice satellite cells and Mouse Ric10 and human myogenic cell clone Hu5KD3. All cell line studies administered vitamin D in the form of 1,25(OH)_2_ D with the exception of only two studies that have used Elocalcitol. Overall, the cell lines studies have used similar methods to report that VitD had positive effects on the development of muscle fibres in cells in culture, skeletal muscle force and hypertrophy or have impact on the regulation of lipid and mitochondrial muscle metabolism or have a direct effect on glucose metabolism and insulin driven signalling (results are stratified by outcome in Table 1). The most common concentration of 1,25(OH)2 that indicated a positive effect in any of the outcomes investigated was 100 nM (11 out of 18) of VitD or Elocalcitol (Table 1). Mechanisms likely to mediate the effects of VitD on skeletal muscle function and energy metabolism are: increased expression of myogenic regulatory factors (such as ↑ MYOD, MYOG, MYC2, skeletal muscle fast troponin I and T, MYH1, IGF1 IGF2, FGF1 and 2, BMP4, MMP9 and FST; increased protein synthesis signalling via AKT, mTOR and GSK3B; increased mitochondrial oxygen consumption rate via mitochondrial gene expression and lipolytic genes (ATGL and CGI-58); increased GLUT4, GLUT1 translocation, insulin receptor expression glucose uptake, 4E-BP1 activation; enhanced Inter-leukin-6 myokine release and inhibition of Interleukin-6 protein which is related with oxidative stress. 

### 3.2. Animal Studies

Six different species of rats and mice were identified in this review (Table 2). Six out of 13 studies used C57BL/6J mice, while two studies used Sprague/Dawley rats, another two studies used Wistar rats and only one study have used each of the following species: A/J mice or p62 deficient mice or the Otsuka Long-Evans Tokushima sedentary fatty rats. The majority of animal studies (six out of 13) have used the most common active form of vitamin D: 1,25(OH)_2_D, while other studies have used 25(OH)D or native cholecalciferol or alfacalcidol or the authors only reported as Vitamin D (Table 2). Overall, animal studies have found that the rat and mice groups who received VitD treatment isolated or with the diet had less weight gain and adipose tissue mass, attenuating the progression of obesity and preserving skeletal muscle function. Vitamin D groups had also an increase in systemic glucose tolerance and improved muscle insulin signalling. The VitD mechanisms reported to be involved in these outcomes are: reduced activation of NFKB, TNFα, the SCAP/SREBP lipogenic pathway; increased FNDC5 gene expression and muscle irisin levels; VitD also stimulates skeletal muscle differentiation and suppressing muscle catabolic genes (decreased atrogin-1 or MuRF1 expression). Other studies reported a reduction in muscle protein synthesis through activation of eIF2α when rats or mice where VitD deficient. 

#### 3.2.1. In Vitro Cell and Animal Studies

##### Myotube Formation, Muscle Mass, Strength and Force

The ability of the muscle to respond to amino acid and insulin levels reduces with age, which increases anabolic resistance and might negatively influence protein absorption and digestion [50]. Muscle mass starts declining in the fourth decade of life and significant reduction can be observed by around 59 years of age [51]. Skeletal muscle anabolism seems to be enhanced by the effects of VitD and dietary protein [52]. This last study indicates that VitD may have both positive and negative effects on muscle homeostasis, (i.e., muscle regeneration and myofiber maintenance) depending on the dose used.

At the cellular level, some studies have investigated the effects of VitD3 in mice satellite cells and C2C12 and human skeletal muscle cells and human myogenic cell clone cells (Table 1). The studies suggested that treatment with the vitamin stimulates cell differentiation into mature muscle fibres [17,26,27]. In the first two studies [17,26] (Table 1) it was observed an increase in the expression of myogenic regulatory factors after treatment with 100 nM of VitD3 for 1–12 days, while the most recent study [27] demonstrated that a high concentration of VitD3 (1000 nM) for only 24 h had the opposite effects, decreasing the same myogenic regulatory factors, but still inducing hypertrophy in multinucleated myotubes. 

Similar results were observed in animal studies (Table 2), where sedentary fatty rats were treated with a 0.1µg/kg/day VitD analogue-alfacalcidol (ALF) with and without low-intensity aerobic exercise for 6 weeks and the combination of ALF with exercise stimulated skeletal muscle differentiation and suppressed muscle catabolic genes [49]. In another study, mice that were injected with a bolus 1500 IU of VitD3 had lower force in the extensor digitorum longus (EDL; fast-twitch) and soleus (slow-twitch) muscles when compared with higher levels of VitD3 (20,000 IU/kg food) for a longer period (9 weeks). These results confirm that different treatment strategies result in different outcomes, especially considering dose and duration [41]. Interestingly, VitD3 associated with a Mediterranean diet resulted in synergetic effect of muscle fibre hypertrophy when compared with regular diet with VitD3 only for 10 weeks in male rats [40] (Table 2). A similar female mouse model was studied to identify the best dose-effect of VitD3 for skeletal muscle function and the authors found that a low dose (100 IU/day) for 6 weeks significantly decreased maximal DIA force, twitch force and CSA fibres when compared with the other groups that had a higher dose of VitD3 (1000 and 10,000 of VitD3/kg food) [39] (Table 2). Overall, VitD3 treatments had positive effects on the development of muscle fibres cells, skeletal muscle force and hypertrophy in mice. New studies focusing on the effects of the VitD3 in aging animals are required to better distinguish its action and optimal dose–effect for skeletal muscle health and function.

##### Muscle function and protein synthesis

A key regulator involved in musculoskeletal health and function is through the mammalian target of rapamycin (mTOR), which impacts several anabolic processes in skeletal muscle [53]. The mechanism involved is through Akt pathway, which results in increased levels of myogenin and myosin heavy chain, essential for skeletal muscle function [54,55]. In this section we will discuss the evidence of VitD treatment in muscle atrophy prevention and its effects in neuromuscular junction (NMJ) function. Hayakawa et al. found that treatment with 10 nM of VitD3 for 24, 48 or 72 h inhibited expression of muscle atrophy F-box (MAFbx) and muscle RING finger (MuRF1) and ubiquitin ligases involved in the development of muscle atrophy in human myotubes [28] (Table 1). A subsequent study with primary human myotubes has revealed that VitD3 significantly increased the expression of Akt, mTOR and GSK3B in association with insulin, demonstrating an additive effect of VitD3 in protein synthesis signalling and also in the number and size of muscle fibres [17] (Table 1). In this study, the authors have proven that VitD3 in combination with insulin had an additive effect in the rate of protein synthesis in human myotubes [17]. These results confirm the stimulus of protein synthesis and hypertrophic effects of VitD3 in primary human cells, which might result in the prevention of skeletal muscle atrophy in humans. 

In animal studies (Table 2), VitD deficiency has led to detrimental effects in skeletal muscle, mainly resulting in muscle atrophy [43,44,48]. Nakamura et al. have demonstrated that low VitD status resulted in worse mobilization and induced muscle atrophy in mice [44] (Table 2). The last authors have concluded that maintaining sufficient levels of VitD is likely to prevent deterioration and skeletal muscle atrophy [44]. In accordance with this study, Gifondorwa et al. have reported that a VitD deficient diet resulted in detrimental changes in the structure and function of the neuromuscular junction [43]. More recently, Kim et al. have studied an obesity model mouse (p62-deficient) and discovered that VitD3 attenuated the progression of obesity and preserved skeletal muscle function when compared with control group [48] (Table 2). In summary, to date these results validate the likely role of VitD3 in preventing skeletal muscle atrophy and ensuring normal neuromuscular junction function. Despite previous reports, there are still insufficient evidence to establish if higher VitD3 doses are beneficial in the aging process or if the prevention of VitD3 deficiency is enough to preserve skeletal muscle function, protein synthesis and NMJ function. 

##### Mitochondria and Lipid Metabolism 

It is well known that intramuscular fat increases with age, which consequently reduces lean muscle mass used for energy metabolism [5]. Discovering strategies to preserve muscle mass in the elderly population is of public health importance. In this section we will discuss the evidence about VitD3 treatment in regard to mitochondria and lipid metabolism. Ryan et al. were one of the first teams to investigate the effects of VitD3 in fat metabolism in C2C12 myotubes [33] (Table 1). They firstly discovered that low VitD3 treatment (0.1–10 nM/6 days) increased fat droplet accumulation, while higher concentrations (100–10,000 nM/6 days) inhibited fat accumulation [33]. These effects were associated with the regulation of Pparg2 and Fabp4 mRNA resulting in lower adipocytes formation after higher concentrations of VitD3. In accordance with this study, Jefferson et al. have demonstrated that treatment with 100 nM of VitD3 in C2C12 myotubes for 96 h increased *p*Akt expression, total ceramides and diacylglycerol, consequently decreasing the amount of lipid within myotubes [15] (Table 1). These changes in lipid metabolism seems to be connected to mitochondrial function in myotubes, as Schnell et al. have confirmed that 100 nM VitD3 for 24 h had significantly increased mitochondrial function, lipolytic genes (ATGL and CGI-58) and oxygen consumption rate (OCR) [34] (Table 1). Similar outcomes were observed by Chang and Kim, as they found a significant increase in ATP levels and mitochondrial function gene expression after 100 nM of VitD3 treatment for 24 h, resulting in a protective effect on muscle fat accumulation and mitochondrial disfunction in C2C12 myotubes [12] (Table 1). 

In this context, the majority of animal studies (Table 2) have focused on the effects of VitD3 on body weight gain using a high or low-fat diet [13,14,42]. Alkharfy et al. found that mice treated with 150 IU/kg/day of VitD3 associated with a low or a fat diet for 16 weeks gained less weight as compared with controls group (without VitD3–6.8% vs. 28.7%). In addition, VitD3 attenuated muscle structure abnormalities caused by a high fat diet [42] (Table 2). Another study has demonstrated similar results after VitD3 treatment also associated with a high fat diet, including less body weight and adipose tissue possibly due to the activation and increase in UCP3 in the muscles [14]. UCP3 is part of the family of uncoupling proteins which mediates energy expenditure via mitochondrial proton leak and lipid metabolism [56]. Interestingly, a significant increase in weight gain, fat mass and intramyocellular lipids in a VitD deficient old rat’s model was also observed [45]. This last study has confirmed a reduction in protein synthesis through the activation of Eif2*a* in rats with a VitD deficient status and these changes were restored by VitD3 [45] (Table 2). Most of scientific data in animal models and cell culture has confirmed the effects of VitD3 regulating lipid and mitochondrial muscle metabolism. More studies are required to prove that VitD supplementation can actually assist preventing obesity or weight gain in humans.

##### Glucose and Insulin Metabolism 

In a healthy condition, skeletal muscle is accountable for ~85% of whole-body insulin-mediated glucose uptake, confirming its importance to insulin resistance development [57]. Recently increasing interest about the role of VitD deficiency and the association with hyperglycaemia and diabetes has been evident [58]. It has been suggested that VitD plays a significant role increasing translocation of the glucose transporter, GLUT4 to the plasma membrane; however, scientific studies are still limited [58]. In this section, we will discuss the current evidence of the effects of VitD in glucose metabolism and insulin signalling. 

Cell culture studies using C2C12 myotubes found a significant increase in the GLUT4 translocation and glucose uptake following 2 h of VitD3 (25 and 50 nM) associated with insulin treatment [35] (Table 1). In this case, the mechanism involved was by SIRT1 activation, with subsequent IRS1 increased phosphorylation [35]. In a model of old diabetic male rats, the treatment with 12 µg/kg of VitD3 for 2 weeks have helped to reverse metabolic changes caused by diabetes, such as decreased insulin receptor expression, increased glycogenolysis and decreased glycogenesis process in skeletal muscle [47] (Table 2). In agreement with these results, Benetti et al. have treated mice with a control or high fat-high sugar diet with or without 7 µg/kg VitD3 for 2 months and they observed that VitD3 group have gained less weight than did the control group (Table 2). These authors also reported that VitD3 treatment increases systemic glucose tolerance and restores impaired muscle insulin signalling [13]; however, it is still unclear whether this association reflects a causal relationship or not. Overall, it appears that VitD3 has a direct effect on glucose and insulin metabolism in cell models in vitro and animal models. Preliminary evidence suggests that VitD3 has the potential to be a therapeutic target, possibly by improving the metabolic control in hyperglycaemia and diabetes conditions. However, clinical studies are necessary to investigate and clarify the precise molecular mechanisms and pathways by which VitD3 acts on glucose and insulin signalling and how it is related to skeletal muscle function. 

##### Oxidative Stress, AGES

Oxidative stress can be described as an imbalance between the level of antioxidant capacity and the production of reactive oxygen species (ROS). Reactive oxygen species have been associated with a wide variety of conditions, such as obesity, hypertension, hyperglycaemia and dyslipidaemia [59]. Vitamin D deficiency and advanced glycation end products (AGEs) are found to be associated with the development of obesity, type 2 diabetes and sarcopenia [60,61]. Advanced glycation end products are a result of reactions of carbohydrates with proteins and its production is found to be higher in elderly and diabetic population, affecting bones and muscle tissue [11,62]. Interestingly, Tanaka et al. observed an increase in the expression of type 1 collagen and the reduction of AGEs production after 48 h of VitD3 in C2C12 myoblasts [37] (Table 1). They also reported that AGEs have suppressed the expression of markers of differentiation in myoblasts (such as MyoD and myogenin) [37]. More recently, Chang and collaborators have reported a decrease in muscle oxidative stress, in lipid peroxidation, in intracellular damage and also in cellular death after 24 h of 1, 10 and 100 nM of VitD3 treatment in C2C12 myotubes [11] (Table 1). In accordance with previous studies, more recently Kim and colleagues have investigated the effects of VitD3 in an obesity mouse model (Table 2). They have reported that 1000 IU VitD3/kg/day for 10 weeks associated with exercise have reduced the weight gain, improved blood glucose levels and decreased spleen mass when compared to control; however, the same effects were not observed in the group that received VITD3 only [48] (Table 2). In this case, it seems that it is the training that is the most important factor that delays the accumulation of AGEs. A subsequent study with adult male rats has revealed that VitD3 treatment increased the gene expression of FNDC5 and muscle irisin levels which are responsible for prevention of weight gain and increase in UCP1 in mitochondria [46] (Table 2). Taken together, to the best of our knowledge, the results suggest that VitD3 might have beneficial effects on the reduction of ROS, on positive mitochondrial changes and on prevention of AGEs. In other words, VitD3 might be useful in the treatment of health complications related to the aging process and further studies should investigate its application in animal and clinical studies. 

### 3.3. Human Studies 

#### Muscle Mass, Strength and Function

Low total serum of [25(OH)D] seems to be linked to the aging process and also to the reduction in muscle performance [7]. In this section, we will discuss data available on the topic and summarize the observed effects of VitD3 supplementation on skeletal muscle function at the three levels of interventions. 

Positive relationships have been seen by Dhesei and colleagues [68] who found that a single intramuscular injection of 600,000 IU ergocalciferol in elderly people with VitD deficiency at baseline resulted in a significant clinical benefit on functional performance, reaction time and balance, but not on muscle strength (evaluated by the quadriceps strength) (Table 3). This suggests that the intervention improves neuromuscular or neuroprotective function, which may in part explain the mechanism whereby VitD could contribute to reducing the incidence of falls and fractures of the elderly. Interestingly, a study of old pre-sarcopenic Lebanese population reported that only muscle mass increased significantly after 6 months supplementation with 10,000 IU VitD3 3x/week (Table 3). They did not find significant effect on muscle strength measured by handgrip strength [63]. Positive results were also seen in the study of Ceglia et al. [66], in which they observed that at 4 months after daily supplementation with 4000 IU of VitD3 in insufficient [25(OH)D] older women, changes in [25(OH)D] level were strongly associated with change in intramyonuclear VDR in muscle tissue samples (r = 0.87, P < 0.001). In addition, the most pronounced group difference was seen in type II fibres (Table 3). Moreover, significant increase in total (type I + type II) fibre cross-sectional area (FCSA) was seen in the VitD3 group (P = 0.048). Fibre type-specific analyses revealed a significant increase in VDR-positive myonuclei in type II fibres after supplementation (P = 0.002) [66] (Table 3). These findings are consistent with the concept that supplementation with VitD3 may promote an increase in VDR content in myocytes and it would therefore sustain the positive clinical effects on muscle mass in this frail population at increased risk for disability. Corroborating the results, Sorensen and col. [69] in 1979 and Sato et al. [70] in 2005 reported similar outcomes. The first team of authors [69], treated 11 ageing osteoporotic women with daily 1–2 µg of VitD analogue 1 alpha-hydroxycholecalciferol and 1 g of calcium for 3–6 months and collected muscle biopsies (Table 3). Fibre composition revealed that supplementation induced an increase in type IIa fibres as well as cross-sectional area. Almost 3 decades later, the second team mentioned above [70] treated half of the 96 elderly women with poststroke hemiplegia that were VitD deficient at baseline with 1000 IU of ergocalciferol daily for 2 years. They showed increases in the relative number and in the size of type II muscle fibres and improved muscle strength in the VitD-treated group (Table 3). A recent study [3] observed an improvement in relaxed multifidus muscle thickness at L2/L3 vertebral levels after monthly dose of 50,000 IU of VitD3 of older adults with symptomatic knee osteoarthritis at the end of the 2 years study (Table 3). A systematic review with meta-analysis on the effects of VitD supplementation on muscle function of elderly participants (mean age: 61.1 years) reported a small but significant positive effect of intervention on global muscle strength, but not on muscle mass or power [71]. 

On the other hand, eight out of the 12 evaluated studies on human have reported contradictory results (Table 3). Seven of these eight articles [1,20,21,64,65,67,72] studied older participants with insufficient or deficient total serum [25(OH)D] at baseline; supplementation was predominantly with VitD3 at a range from 400–2000 IU daily until 20,000 IU 1/week or even single dose of 300,000 IU calciferol; follow-up period was from 2 months to 1 year; and sample size ranged from 8–208 participants per studied group (Table 3). Neither of these studies reported any significant change in muscle outcomes at the end of the study compared to the placebo group. Bislev et al. [9] conducted a study with only postmenopausal women VitD deficient providing 2800 IU VitD3 daily for 3 months. Supplementation did not improve any of the studied outcomes (handgrip, knee flexion strength, timed up and go test, lean mass, physical performance); in fact, the three first measurements described decreased over time in this group (Table 3). However, this study has a particular potential limitation as the initial factorial design involved half of the participants in each arm to be treated with valsartan 80 mg/day for the first two weeks in order to investigate the PTH response to treatment with an angiotensin 2 receptor blocker; however, there was no effect of the angiotensin 2 receptor blocker on PTH levels and no interaction between valsartan and VitD3. 

All intervention groups that received VitD3 had their dosing regimen sufficient (regardless of frequency of intake) to increase serum [25(OH)D] to normality and it corresponds to the concentration that previous studies have reported to be associated with better muscle function; however, studies evaluated cross-sectional and longitudinal associations [73,74,75] (Table 3). Kuchuk and colleagues [76] proposed that physical performance in older persons are likely to improve when serum [25(OH)D] levels raise above 50–60 nmol/L. However, most of the studies in this review showed normalization of [25(OH)D] status after a wide range of different intervention methods (in terms of dosage and frequency of intakes), but still no significant effects on muscle mass were observed when compared to participants in the placebo group (Table 3). In addition, to that, our study also showed inconsistency to a meta-analysis of randomised controlled trials that evaluated the effects of VitD3 supplementation on muscle function and point out that it may be more beneficial to muscle strength if total serum [25(OH)D] at baseline was < 30 nmol/L [71]. Previous meta-analysis of trials concluded either a small beneficial effect (41, 50) or no effect (51) of VitD3 supplementation on muscle mass or function. 

The current review may have revealed predominance of null effects of VitD3 on muscle due to several reasons: 1. reviewed studies are highly heterogenous in terms of sample size, dosage, frequency of intakes and duration; 2. many included studies had modest sample size and this may be a plausible reason why most of them had limited power to detect meaningful effects between groups; 3. some studies cannot comment on the degree to which these findings may vary in men or women; 4. it seems plausible that calcium supplementation needs to be added to VitD3 in order to produce a beneficial effect on physical performance as suggested by Pfeifer and colleagues [77] in a study with 242 elderly individuals (mean 77 ± 4 years), all serum [25(OH)D] levels below 78 nmol/L where participants received either 1000 mg of calcium or 1000 mg of calcium plus 800 IU of VitD3 per day over a treatment period of 12 months. There, combined calcium and VitD3 supplementation proved to be superior to calcium alone in reducing the number of falls and improving muscle function. However, this needs to be confirmed in further clinical trials; 5. physical activity decreases with age, negatively affecting muscle mass and contractile function and predisposing to weight gain, mainly as fat mass. Increased fat mass promotes insulin resistance and can produce a direct catabolic effect on skeletal muscle. It is possible that a positive treatment effect on muscle protein synthesis would have been observed in exercising muscles. 6. The studies included in this review did not measure the bioavailable free form of VitD (free 25(OH)D), which has been correlated with many biological actions of VitD3 [78]. 

To the best of our knowledge, this is the first review that comprehensively assess the evidence for the impact of VitD on skeletal muscle function in an aging population at the molecular and clinical level, citing results from studies in vitro, in vivo and clinical research. In addition, the authors have followed the PRISMA protocols for a systematic review to guarantee a standardisation of the research and to avoid bias. In this review, we have included high quality RCTs (10 out of 13 studies were classified as excellent using PEDro scale) [79]. On the other hand, we have included only studies that were published in English, which might have implications for language bias and it represents a limitation. In addition, we have found that the studies in each area (cells, animals and humans) have heterogeneity of species, especially considering cell lines, animals and different population included in RCT. Other sources of heterogeneity identified in the trials are: the participant characteristics, VitD form, dose and protocol, duration of the intervention, RCT variables such as and strength and power testing measures. In this review, we did not investigate the impact of VitD on subsequent falls reduction. Further studies are therefore required to determine the effect of VitD on other parameters.

Key nutrient supplementation in older adults is of consideration in the prevention of sarcopenia, especially because it is a simple, low-cost treatment approach without major side effects. Vitamin D supplementation for skeletal muscle health, function focused on the aging process have recently received increased attention. Our objective was to comprehensively review the literature and summarize the current knowledge on this topic, resulting in a better understanding of the potential VitD effects when analysed from all the perspectives: cellular, animal and clinical. Since medical guideline recommendations for health and prevention of diseases are based on adequate total serum levels of 25(OH)D, cellular studies can actually evaluate the effect of various doses of 25(OH)D that mimic whole body circulating concentrations of VitD. The main findings in cellular and animal models reported in this review include beneficial regulation of muscle formation, mass, strength and force by VitD. Cellular and animal studies also found beneficial effects of VitD on mitochondrial and lipid metabolism, glucose regulation and insulin metabolism and oxidative stress. All these effects might be applicable as a therapeutic for the prevention and/or treatment with associated health complications such as metabolic disease, diabetes, obesity another chronic conditions. Based on these previous molecular studies included in this review, future studies now can focus on the attempt to replicate the treatment with VitD focusing on the possible regulation of expression of proteins involved in the skeletal, lipid and glucose metabolism, such as AMPK, MuRF1, SIRT1, UCPs, IGFs, AKT, mTOR, GSK3B, FOXO1, GLUT4 in an aging model. Another important consideration for future studies is that the majority of studies included in this review, investigated older participants with insufficient or deficient total serum [25(OH)D] at baseline. Benefits for skeletal muscle function regarding a higher concentration of serum VitD after supplementation remain to be reported. Lack of consistency across the evaluated studies resulted in inconsistent evidence that supplementation with VitD3 has positive effects on muscle mass or function in older people. Further well-designed, large clinical trials and meta-analyses are required to determine outcomes more precisely in order to determine whether VitD3 alone or combined with other supplements or exercise programmes impact positively on i. muscle fibre size; ii. muscle performance; iii. VDR concentration; iv. the signalling pathways that are involved.

## 4. Conclusions

The purpose of this review was to summarize the effects of VitD3 supplementation on skeletal muscle in cell, animals and in an aging population. Increasing total serum [25(OH)D] levels from insufficiency/deficiency status to normality does not appear to benefit muscle function, power or mass in older adults. Our review suggests that improvements in muscle performance in older adults cannot be guaranteed from VitD3 supplementation alone, at least over a short timeframe. Therefore, a combination of exercise, VitD supplementation and longer-term interventions may be more effective in increasing skeletal muscle health. Well-designed long duration double-blinded trials, standardised VitD3 dosing regimen, larger sample sized studies and standardised measurements may be helpful to determine favourable outcomes and future recommendations.

## Figures and Tables

**Table 1:** Overview of the effects of vitamin D (VitD) in skeletal muscle cells (*n* = 18 in vitro studies) stratified by outcome.

The authors, Year	Cell Line/Type	Maturation	VitD Dose, Form, Time	Significant Findings and Effects	Comments
Myotube formation, muscle mass, strength and force					
Braga et al., 2017 [26]	Mice satellite cells	Myoblasts	100 nM, 1,25(OH)_2_D, 1–12d	↑ MYOD, MYOG, MYC2, skeletal muscle fast troponin I and T, MYH1, IGF1 IGF2, FGF1 and 2, BMP4, MMP9 and FST.	VitD3 promoted a robust myogenic effect on satellite cells responsible for the regeneration of muscle after injury or muscle waste.
Romeu et al., 2019 [17]	HSMM	Myoblasts and myotubes	100 nM, 1,25(OH)_2_D, 48 h/or 5 d	↑ differentiation by altering the expression of myogenic regulatory factors. ↑ protein synthesis signalling and synthesis (AKT, mTOR, GSK3B); ↑ OCR in myoblasts and myotubes.	At day 6, there were notably higher number and diameter of myotubes per field in VitD3 group when compared with vehicle group.
Hosoyama et al., 2020 [27]	Mouse Ric10 and human myogenic cell clone Hu5KD3	Myoblasts and myotubes	1000 nM, 1,25(OH)_2_D, 24 h	Induced hypertrophy of multinucleated myotubes by stimulating protein anabolism	↓ expression of MRFs, Myf5 and myogenin in proliferating myoblasts. High concentration of VitD reduced myoblast-to-myoblast and myoblast-to-myotube fusion through the inhibition of Tmem8c (myomaker) and Gm7325 (myomerger).
Muscle function and protein synthesis					
Hayakawa et al., 2015 [28]	HSMM	Myotubes	10 nM, 1,25(OH)_2_D, 24/48/or 72 h	↑ interleukin-6 expression and inhibited expression of TNF-α, MAFbx, MuRF1 and ubiquitin ligases involved in muscle atrophy after VitD3 treatment.	VitD3 suppresses muscle degradation and its likely to be involved in the regulation of apoptosis, insulin responsiveness and myogenesis in skeletal muscle.
Van der Meijden et al., 2016 [29]	C2C12	Myoblasts and myotubes	0/400/1000/or 2000 nmol/L, 1,25(OH)_2_D, 24 h	Myoblasts and myotubes were able to convert 25(OH)D3 to 24,25(OH)2D3 locally (skeletal muscle); ↑ VDR and MHC mRNA expression.	Skeletal muscle is not only a direct target for VitD3 metabolites, but is also to its precursor.
Antinozzi et al., 2017 [30]	Human fetal skeletal muscle cells	Myoblasts	10/or 100 nM, Elocalcitol, 1–24 h	Elocalcitol exerted an I-like effect, promoting GLUT4 re-localization in Flotillin-1, Caveolin-3 and Caveolin-1 positive sites and mTOR, AKT, ERK, 4E-BP1 activation; it enhanced Interleukin-6 myokine release.	VDR agonists as elocalcitol may be therapeutic tools for skeletal muscle integrity/function maintenance, an indispensable condition for health homeostasis.
Hirose et al., 2018 [31]	C2C12	Myoblasts	1000 and 10,000 nM, 1,25(OH)_2_D, 24 h	↓ FOXO1-mediated; glucocorticoid-induced gene expression of atrogin 1 and cathepsin L	VitD3 may prevent muscle atrophy via the FOXO1-mediated pathway in muscle cells.
Arakawa & Wagatsuma, 2020 [32]	C2C12	Myotubes	100 nM, 1,25(OH)_2_D, 24 h	Enhanced agrin- induced AChR clustering in myotubes compared to treatment with agrin alone.	VitD3 -VDR signalling may regulate rapsin expression, resulting in the up-regulation of agrin- induced AChR clustering.
Mitochondria and lipid metabolism					
Ryan et al., 2013 [33]	C2C12	Myotubes	0.1–10000 nM, 1,25(OH)_2_D, ≤ 6 d	Low physiological concentrations (10K13 and 10K11 M) of VitD3 increased fat droplet accumulation; high physiological (10K9 M) and supraphysiological concentrations (R10K7 M) inhibited fat accumulation.	Low VitD concentration was associated with a sequential up-regulation of Pparg2 and Fabp4 mRNA, indicating formation of adipocytes, whereas higher concentrations reduced all these effects.
Jefferson et al., 2017 [15]	C2C12	Myotubes	100 nM, 1,25(OH)_2_D, 96 h	↑ insulin-stimulated *p*Akt; ↑ total ceramides and DAG in a subspecies specific manner; ↓ the proportion of lipid within myotubes.	VitD3 altered myocellular lipid partitioning and lipid droplet packaging, lipid turnover and partially explained improvements in insulin sensitivity.
Chang & Kim, 2019 [11]	C2C12	Myotubes	100 nM, 1,25(OH)_2_D, 24 h	↑ ATP levels and mitochondrial function gene expression: CPT1, PPARα, VLCAD, LCAD, MCAD, UCP2 and UCP3. ↑ SIRT1 mRNA expression, ↑ activation of AMPK and SIRT1	Protective effect of VitD3 on muscle fat accumulation and mitochondrial dysfunction ↑ mtDNA, NRF1, PGC-1α and mitochondrial transcription factor A (Tfam) in C2C12 myotubes.
Schnell et al., 2019 [34]	C2C12	Myotubes	100 nM, 1,25(OH)_2_D, 24 h	↑ mitochondrial function in myotubes (↑ lipolytic genes ATGL and CGI-58, OCR	↑ mRNA expression of triglyceride synthesizing genes DGAT1 and DGAT2; in part mediated by Perilipin-2
Glucose and insulin metabolism					
Manna et al., 2017 [35]	C2C12	Myotubes	25 or 50 nM, 1,25(OH)_2_D, 2 h	VitD3 + insulin ↑ GLUT4 translocation and glucose uptake	VitD3 ↑ glucose consumption by inducing SIRT1 activation, which in turn increases IRS1 phosphorylation and GLUT4 translocation in myotubes.
Tamilselvan et al., 2013 [36]	L6	Myotubes	100 nM, 1,25(OH)_2_D, 24 h	↑ GLUT1, GLUT4, VDR and insulin receptor expression.	Potential antidiabetic role of VitD in regulation of expression of the glucose transporters in muscle cells.
Antinozzi et al., 2019 [18]	Human fetal skeletal muscle cells	Myoblasts	10 and 100 nM, Elocalcitol, 15 min	Elocalcitol induced GLUT4 protein translocation likely in lipid raft microdomains; rapid IRS1 phosphorylation; inflammatory myopathy subjects, had VitD deficiency and a high lipidemic and resistin profile, possibly increasing the risk to develop metabolic diseases.	Elocalcitol might be a therapeutic tool for skeletal muscle integrity/function maintenance and important for health homeostasis.
Oxidative stress and AGES					
Tanaka et al., 2014 [37]	C2C12	Myoblasts	0.1 nM, 1,25(OH)_2_D, 48 h	↑ expression of type 1 collagen; AGE2 and AGE3 suppressed the expression of MyoD, myogenin and OGN. 1,25D blunted the AGEs’ effects.	VitD3 may rescue the AGEs-induced sarcopenia as well as–suppressed osteoblastic differentiation via OGN expression in myoblasts.
Chang et al., 2019 [11]	C2C12	Myotubes	1, 10 and 100 nM, 1,25(OH)_2_D, 24 h	↑ mtDNA, PGC1α, NRF1, Tfam, NRF2, NAD levels, activities of AMPK, SIRT1, expression of HMOX1 and TXNRD1.	↓ muscle oxidative stress, lipid peroxidation, intracellular damage and cell death
Nonaka et al., 2020 [38]	C2C12	Myotubes	0/0.1/1/or 10 nM, 1,25(OH)_2_D, 4 d	VitD3 inhibited increases in Interleukin-6 protein, suggesting that VitD3 inhibits inflammation in muscle cells.	VitD3 can prevent or improve sarcopenia, which is associated with interleukin-6.

VitD3 = cholecalciferol; mtDNA = Mitochondrial DNA; PGC1α = proliferator-activated receptor gamma coactivator 1-alpha; NRF1 = Nuclear Respiratory Factor 1; Tfam = Mitochondrial transcription factor A; NRF2 = Nuclear factor erythroid-2-related factor 2; NAD = Nicotinamide adenine dinucleotide; AMPK = 5’ adenosine monophosphate-activated protein kinase; SIRT1 = Sirtulin 1; HMOX1 = Heme Oxygenase 1; TXNRD1 = Thioredoxin Reductase 1; ATP = Adenosine triphosphate; CPT1 = carnitine palmitoyl transferase 1; PPARα peroxisome proliferator-activated receptor α; VLCAD = very long-chain acyl-CoA dehydrogenase; LCAD = Long-chain acyl-CoA dehydrogenase; MCAD = medium-chain acyl-CoA dehydrogenase; UCP2 = uncoupling protein 2; UCP3 = uncoupling protein 3; FOXO1 = Forkhead box protein O1; Atrogin-1 = muscle-specific F-box protein; Cathepsin L = lysosomal endopeptidase enzyme; AKT = Protein kinase B; mTOR = mammalian target of rapamycin; GSK3B = Glycogen synthase kinase 3 beta; OCR = oxygen consumption rate; ATGL = Adipose triglyceride lipase; CGI-58 = Comparative Gene Identification (activator of triglyceride hydrolases and as acyl-CoA); DGAT1 = Diacylglycerol O-Acyltransferase 1; DGAT2 = Diacylglycerol O-acyltransferase 2; Perilipin-2: Adipose differentiation-related protein; GLUT = Glucose transporter (type 1, 4); IRS1 = Insulin receptor substrate 1; MYOD = myoblast determination protein 1; MYOG = Myogenin; MYC2 = transcription factor; MYH1 = Myosin heavy chain 1; IGF1 and 2 = Insulin-like growth factor; FGF1 and 2 = acidic fibroblast growth factor; BMP4 = Bone morphogenetic protein 4; MMP9 = 92 kDa type IV collagenase; FST = gene encoded Follistatin; VDR = Vitamin D receptor; MHC = Major histocompatibility complex; AGEs: advanced glycation end products; OGN = Osteoglycin; ERK = Extracellular signal-regulated kinase; TNF-α = of tumor necrosis factor alpha; MAFbx = muscle atrophy F-box; MuRF1 = muscle RING-finger protein-1; Pparg2 = Peroxisome proliferator-activated receptor gamma; Fabp4 = Fatty Acid-Binding Protein 4; AChR = acetylcholine receptor; LPS = Lipopolysaccharide; MRFs = myogenic regulatory factors; Myf5 = Myogenic factor 5. ↑ = statistically increased (between groups analysis); ↓ = statistically decreased (between groups analysis).

**Table 2:** Overview of the effects of vitamin D (VitD) in skeletal muscle (*n* = 13 animal studies) stratified by outcome.

The authors, Year	Specie, N, Age	Groups (VitD Form and Dose), Time	Significant Findings and Effects	Comments
Myotube formation, muscle mass, strength and force				
Ray et al., 2016 [39]	A/J mice, *n* = 20 (5 per group), 4- or 12-weeks old female	G1 (Low, 100 IU); G2 (Reference, 1000 IU); G3 (10,000 IU 1,25(OH)_2_D/kg), 6 weeks	Low VitD3 group had ↓ maximal diaphragm (DIA) force, twitch force and fiber CSA (26%, 28% and 10% respectively).	Potential role of VitD3 in regulating DIA development and insulin sensitivity.
Trovato et al., 2018 [40]	Sprague/Dawley rats, *n* = 28 (4 per group), 7–9 weeks old male	RD (regular diet); R-DS (R + 4000 IU/KgVitD); RDR = regular diet without VitD; HFB-DS (high-fat diet +VitD); HFB-DR (high-fat w/o VitD); HFEVO-DS (high-fat + VitD); HFEVO-DR (high-fat + w/o VitD),10 weeks	Muscle fibres of high fat diet + VitD3 rats were hypertrophic comparing to those of regular diet + VitD3.	VitD associated with a Mediterranean diet showed trophic action on the muscle fibres.
Hayes et al., 2019 [41]	C57BL/6J mice *n* = 32, 8 weeks old	Control (standard chow + 1000 IU/kgVitD3); High (same diet with 20,000 IU/kg of 25-hydroxyvitamin D); YEAR (injected bolus of 1500 IU25-hydroxyvitamin D) 4 weeks	YEAR ↓ forces in both muscles compared to High, as well as lower force during fatigue and early recovery.	Mice ingesting the same amount of food + VitD3 over four weeks did not demonstrate the same detrimental effects.
Muscle function and protein synthesis				
Alkharfy et al., 2012 [42]	C57BL/6J mice, *n* = 44 (11 pergroup), 4–5 weeks old	Low fat diet (LFD); High fat diet (HFD) with and without 150 IU/kg/day 1,25(OH)_2_D, 16 weeks	HFD with VitD3 showed less weight gain as compared to controls (6.8% vs. 28.7%, respectively).	Muscle structural abnormalities caused by HFD were attenuated by VitD3; tissues have regained their normal structural appearance.
Gifondorwa et al., 2016 [43]	C57BL/6J mice, *n* = 24 (6 per group), 3 weeks old male	G1 (VitD+/Ca+: 1000 IU/kg/0.50%); G2 (VitD+/Ca-1000 IU/kg/0.01%); G3 (VitD-/Ca+: 0 IU/kg/0.47%); G4: (Vit. D2-/Ca-: 0.02%; 0 IU/kg), 9 weeks	VitD3 lead to metabolic changes, NMJ-related and protein chaperoning and refolding genes.	VitD deficient or a VitD and Ca^+2^ deficient diet resulted in detrimental changes in the structure and function of the NMJ.
Nakamura et al., 2020 [44]	C57BL/6J mice, *n* = 5 per group, 9 weeks old	Standard (S) diet, High 1,25(OH)_2_D diet = (VitD3 and Ca: 0.47%, P: 0.3%,) and Low 1,25(OH)_2_D diet = (Ca: 2%, P: 1.25%), 4–8 weeks	VitD low status worsens immobilization-induced muscle atrophy in mice. Mice globally lacking VDR exhibited more severe muscle atrophy following limb immobilization than controls.	Maintaining VitD status at an appropriate level before injury or decline in physical activity is likely crucial to prevent deterioration and muscle atrophy.
Mitochondria and lipid metabolism				
Fan et al., 2016 [14]	C57BL/6J mice, *n* = 15, 8 weeks old male	NFD (control normal-fat diet); HFD (high-fat diet); HFVD (45 kcal % fat; 50 μg/kg body weight/d 25-hydroxyvitamin D),9 weeks	HFVD ↓ body weight and adipose tissue weight and ↑ expression of UCP3 compared to the other groups.	Changes in the expression of genes correlated with VitD3/VDR. VitD3/VDR inhibits weight gain by activating UCP3 in the muscles.
Chanet et al., 2017 [45]	Wistar rats, *n* = 50, 15 months old male	Control (1 IU VitD3/g); VitD-depleted [VDD, diet 0 IU 1,25(OH)_2_D)], 6 months	Weight gain was associated with ↑ in fat mass (+63%, p < 0.05), intramyocellular lipids (+75%, p < 0.05) in VDD.	VitD3 deficiency in old rats ↑ adiposity and leads to reduced muscle protein synthesis through activation of eIF2α. These disorders are restored by VitD3.
Glucose and insulin metabolism				
Benetti et al., 2018 [13]	C57BL/6J mice, *n* = 40, 4 weeks old male	Control or High Fat-High Sugar (HFHS) diet for 4 months; Then, another subset of animals: 1,25(OH)_2_D (7 μg/kg^−1^, 3 times a week) for 2 months	VitD3 ↓ body weight and ↑ systemic glucose tolerance. VitD3 restored the impaired muscle insulin signalling and reverted myosteatosis diet-induced.	VitD3 ↓ activation NFKB and ↓ TNFα, ↓ activation of the SCAP/SREBP lipogenic pathway, ↓ CML protein adducts and RAGE expression.
Nadimi et al., 2019 [46]	Sprague-Dawley rats, *n* = 36, adult male	G1 (healthy control); G2 (healthy receiving sesame oil as placebo); G3 (diabetics receiving sesame oil as placebo); G4 (diabetics treated with 4300 IU/kg/week native cholecalciferol), 4 weeks	VitD ↑ FNDC5 gene expression and muscle irisin levels.	Potential therapeutic effect of VitD3 supplementation for diabetes mellitus.
Xavier et al., 2012 [47]	Wistar rats, *n* = 6–8 per group, 6 months old male	12 μg/kg VitD3 to (a) control; (b) diabetic; (c) insulin-treated diabetic; (d) 1,25(OH)_2_D -treated diabetic; (e) curcumin-treated diabetic rats, 2 weeks	↑ β2-adrenoceptor and CREB gene expression were observed in the diabetic group and ↓ insulin receptor expression, resulting in ↑ glycogenolysis, gluconeogenesis and ↓ glycogenesis in the muscles.	These results were reversed with VitD3 and curcumin treatment. VitD3 and curcumin might help in the management of peripheral complications associated with diabetes.
Kim et al., 2020 [48]	p62-deficient mice, *n* = 10 per group, 24 weeks old male	Control (no treatment); cholecalciferol = 1000 IU VitD3/kg/d), RT = ladder climbing, 3 times per week or combined treatment, VRT = VitD3 + RT), 10 weeks VitD3	Total body mass increased in all groups, but fat mass increased only in control group. Loss of skeletal muscle function was reported only in control group. Improved blood glucose levels and ↓ spleen mass was reported in RT and VRT compared to control.	VitD3 attenuated the progression of obesity and preserved skeletal muscle function.
Akagawa et al., 2018 [49]	Otsuka Long-Evans Tokushima sedentary fatty rats (8–10/group) 20 weeks old	ALF (alfacalcidol 0.1 μg/kg/day); Exe (low-intensity aerobic exercise training); Comb (alfacalcidol + low-intensity aerobic exercise training); T2DM control group, 2 or 6 weeks	ALF, Exe and Comb treatments for 2 and 6 weeks recovered the CSA compared to Control. ALF and Comb for 6 weeks increased femoral BMD compared to Control. ALF or Exe monotherapy significantly decreased Atrogin-1 or MuRF1 expression after 2 weeks. After 6 weeks, ALF and Comb decreased Atrogin-1 and REDD1.	A combination of ALF and Exe improved CSA from the early phase of treatment by stimulating skeletal muscle differentiation and suppressing muscle catabolic genes. Improvements in blood glucose, BMD and CSA were observed as long-term effects of the combination therapy.

VitD3 = cholecalciferol; NMJ = neuromuscular junction; NFKB = major transcription factor; TNF-α = of tumor necrosis factor alpha; SCAP/SREBP = cleavage-activating protein; CML = carboxymethyllysine; RAGE = receptor for advanced glycation end products; UCP3 = uncoupling protein 3; VDR = Vitamin D receptor; CSA = cross-sectional area; DIA = diaphragm Eif2α = Eukaryotic Initiation Factor 2; FNDC5 = fibronectin type III domain containing 5; CREB = cellular transcription factor; MuRF1 = muscle RING-finger protein-1; REDD1 = regulated in development and DNA damage responses ; BMD = bone mineral density; Ca^+2^ = calcium ion. ↑ = statistically increased (between groups analysis); ↓ = statistically decreased (between groups analysis).

**Table 3:** Overview of the effects of vitamin D (VitD) in skeletal muscle in an aging population (*n* = 13).

The authors, Year	Male/ Female (n)	Baseline Serum [25(OH)D]	N	Dose (IU)	Type	Frequency	Duration (weeks)	INTERVENTION Outcomes	N	PLACEBO Outcomes	Comments
El Hajj et al., 2018 [63]	59/56	Deficient	60	10000	Cholecalciferol	3x/week	24	↔ Handgrip (kg) ↑ Muscle mass (kg)	55	↔ Handgrip (kg) ↔ Muscle mass (kg)	Serum [25(OH)D] had significantly change at the end of the study between groups.
Bislev LS et al., 2018 [9]	0/81	Deficient	40	2800	Cholecalciferol	daily	12	↓ Handgrip (N) ↓ Knee flexion 60° strength (N) ↔ Lean mass (kg) ↑ Timed up and go test (sec) ↔ Physical performance (MET score)	41	↑ Handgrip (N) ↑ Knee flexion 60° strength (N) ↔ Lean mass (kg) ↓ Timed up and go test (sec) ↔ Physical performance (MET score)	Serum [25(OH)D] had significantly change at the end of the study between groups.
Shea MK et al., 2019 [1]	64/36	Deficient	47	800–1600	Cholecalciferol	daily	48	↔ Double leg press power W 40% 1RM ↔ Double leg press power W 70% 1RM ↔ Double leg press strength 1RM ↔ Grip strength (kg) ↔ Total lean body mass (kg)	50	↔ Double leg press power W 40% 1RM ↔ Double leg press power W 70% 1RM ↔ Double leg press strength 1RM ↔ Grip strength (kg) ↔ Total lean body mass (kg)	At the 4-mo visit, if a participant from VitD group had serum [25(OH)D] <28 ng/mL, it was given an additional VitD3 capsule (800 IU)/day. To everyone else was given an additional placebo pill. There was only significant change on serum [25(OH)D] at the end of the study between groups.
Vaes AMM et al., 2018 [64] Vaes AMM et al., 2018 [64]	43/32 43/32	Deficient Deficient	24 26	800 400	Cholecalciferol 25-hydroxycholecalciferol	daily daily	24 24	↔ Handgrip (kg) ↔ Timed up and go test (sec) ↔ SPPB total (points 0–12) ↔ Knee extension (Nm) ↔ Knee flexion (Nm) ↔ Total lean mass (kg) ↔ Handgrip (kg) ↔ Timed up and go test (sec) ↔ SPPB total (points 0–12) ↔ Knee extension (Nm) ↔ Knee flexion (Nm) ↔ Total lean mass (kg)	25	↔ Handgrip ↔ Timed up and go test (sec) ↔ SPPB total (points 0–12) ↔ Knee extension (Nm) ↔ Knee flexion (Nm) ↔ Total lean mass (kg)	3 intervention groups: 25(OH)D3, VitD3 and placebo. In both treatment with VitD, serum [25(OH)D] increased.
Grimnes G et al., 2019 [21]	219/192	Deficient	208	20000	Cholecalciferol	1/week	16	↔ Handgrip (kg) ↔ Hip flexion (N) ↔ Biceps flexion (N) ↔ Pectoralis (N)	203	↔ Handgrip ↔ Hip flexion (N) ↔ Biceps flexion (N) ↔ Pectoralis	There was only a change in serum [25(OH)D] at the end of the study between groups.
Van Vliet S et al., 2020 [65]	6/11	Sufficient or Insufficient	9	2000	Cholecalciferol	daily	8	↔ Handgrip (kg) ↔ Myofibrillar protein synthesis rate	8	↔ Handgrip (kg) ↔ Myofibrillar protein synthesis rate	There was only increase in serum [25(OH)D] at the end on the treatment group.
Cuellar WA et al., 2019 [3]	113/104	Deficient to Sufficient	104	50000	Cholecalciferol	1 capsule/ month	96	↔ Trunk muscle size ↔ Chance in thickness of muscle with contraction ↑ Relaxed multifidus muscle thickness (cm) at L2/L3 when adjusted for age + sex + BMI + leg strength	113	↔ Trunk muscle size ↔ Chance in thickness of muscle with contraction ↓ Relaxed multifidus muscle thickness (cm) at L2/L3	Serum [25(OH) D] in the VitD group increased more at the end of the study.
Ceglia L et al., 2013 [66]	0/21	Insufficient	9	4000	Cholecalciferol	Daily	16	↔ 40% and 70% of 1RM average power in knee extension (W) ↑ %∆ in total FCSA (type I + type II) ↔ Type I muscle FCSA (µm^2^) ↔ Type II muscle FCSA (µm^2^) ↑ %∆ in [VDR] ↑ %VDR-positive myonuclei in type II fibres	12	↔ 40% and 70% of 1RM average power in knee extension (W) ↓ %∆ in total FCSA (type I + type II) ↔ Type I muscle FCSA (µm^2^) ↔ Type II muscle FCSA (µm^2^) %∆ in [VDR] %VDR-positive myonuclei in type II fibres	There was a significant increase in serum [25(OH)D] in the vitamin D compared with placebo group at the end of the study.
Latham NK et al., 2003 [67]	114/129	Deficient	108	300000	calciferol	single dose	24	↔Physical component score (mean) ↔ Quadriceps strength (kg) ↔ Timed up and go (sec)	114	↔Physical component score (mean) ↔ Quadriceps strength (kg) ↔ Timed up and go (sec)	The single dose of VitD was effective only in increasing mean [25(OH)D] in the intervention group at the end of the study.
Dhesi JK et al., 2004 [68]	30/108	Deficient	62	600000	ergocalciferol	single intramuscular injection	24	↔ MVC (quadriceps strength, N) ↓ Aggregate functional performance time (sec) ↑ Choice reaction time (sec) ↓ Postural sway	61	↔ MVC (quadriceps strength, N) ↑ Aggregate functional performance time (sec) ↓ Choice reaction time (sec) ↑ Postural sway	Serum [25(OH)D] increased significantly in the end of the study in the treatment group.
Pirotta S et al., 2015 [20]	13/13	Insufficient	13	2000	Cholecalciferol	daily	10	↔ KE 120°/s (N/kg) ↔ KE 180°/s (N/kg) ↔ KE 240°/s (N/kg) ↔Stair clim power (W) ↔Timed up and go test (m/s)	12	↔ KE 120°/s (N/kg) ↔ KE 180°/s (N/kg) ↔ KE 240°/s (N/kg) ↔Stair clim power (W) ↔Timed up and go test (m/s)	At the end of the study, only serum [25(OH)D] increased at the end of the study in the vitamin D group.
Wood AD et al., 2014 [67]	0/265	Insufficient	84 90	400 1000	Cholecalciferol	daily	48	↔ Grip strength (kg) ↔ Grip strength (kg)	91	↔ Grip strength (kg)	3 intervention groups: low VitD3 dose, high VitD3 dose and placebo. Serum [25(OH)D] > 60 nmol/L in both VitD3 groups.

VitD3 = cholecalciferol; ↔ = no statistical difference between groups; ↑ = statistically increased (between groups analysis); ↓ = statistically decreased (between groups analysis); N = newton; MET = metabolic equivalent of task; W = watts; 1RM = 1 repetition maximum; 25(OH)D3 = 25-hydroxycholecalciferol; SPPB = short physical performance battery; Nm = newton meters; BMI = body mass index; %∆ = percent change; FCSA = fibre cross- sectional area; [VDR] = VitD receptor concentration; MVC (maximal voluntary contraction); KE = knee extension.
